# Pathophysiology of Diabetic Retinopathy

**DOI:** 10.1155/2013/343560

**Published:** 2013-01-15

**Authors:** Joanna M. Tarr, Kirti Kaul, Mohit Chopra, Eva M. Kohner, Rakesh Chibber

**Affiliations:** Institute of Biomedical and Clinical Science, Peninsula College of Medicine and Dentistry, University of Exeter, St Luke's Campus, Magdalen Road, Exeter EX1 2LU, UK

## Abstract

Diabetes is now regarded as an epidemic, with the population of patients expected to rise to 380 million by 2025. Tragically, this will lead to approximately 4 million people around the world losing their sight from diabetic retinopathy, the leading cause of blindness in patients aged 20 to 74 years. The risk of development and progression of diabetic retinopathy is closely associated with the type and duration of diabetes, blood glucose, blood pressure, and possibly lipids. Although landmark cross-sectional studies have confirmed the strong relationship between chronic hyperglycaemia and the development and progression of diabetic retinopathy, the underlying mechanism of how hyperglycaemia causes retinal microvascular damage remains unclear. Continued research worldwide has focussed on understanding the pathogenic mechanisms with the ultimate goal to prevent DR. The aim of this paper is to introduce the multiple interconnecting biochemical pathways that have been proposed and tested as key contributors in the development of DR, namely, increased polyol pathway, activation of protein kinase C (PKC), increased expression of growth factors such as vascular endothelial growth factor (VEGF) and insulin-like growth factor-1 (IGF-1), haemodynamic changes, accelerated formation of advanced glycation endproducts (AGEs), oxidative stress, activation of the renin-angiotensin-aldosterone system (RAAS), and subclinical inflammation and capillary occlusion. New pharmacological therapies based on some of these underlying pathogenic mechanisms are also discussed.

## 1. Introduction 

With diabetes now recognised as a global epidemic, the incidence of retinopathy, a common microvascular complication of diabetes, is expected to rise to alarming levels. Diabetic retinopathy is classified into nonproliferative diabetic retinopathy (NPDR) and proliferative diabetic retinopathy (PDR), characterised by the growth of new blood vessels (retinal neovascularization). NPDR is further divided into mild, moderate, and severe stages that may or may not involve the development of a macula diabetic macular oedema (DMO) [1]. The major causes of severe visual impairment are PDR and DMO. Nearly all patients with Type 1 diabetes and >60% of patients with Type 2 diabetes are expected to have some form of retinopathy by the first decade of incidence of diabetes [2, 3].

The risk of developing diabetic retinopathy can be reduced by early detection, timely tight control of blood glucose, blood pressure, and possibly lipids; however, clinically this is difficult to achieve. Laser photocoagulation and vitrectomy are required to treat sight-threatening retinopathy. There is an urgent need to understand how diabetes causes damage to the blood vessels in the eye, to drive the development of new drugs for the treatment of diabetic retinopathy. 

The Diabetes Control and Complications Trial (DCCT) and United Kingdom Prospective Diabetes Study (UKPDS) clinical trials confirmed the strong relationship between chronic hyperglycaemia and the development and progression of diabetic retinopathy, but the underlying mechanism that leads to the development of microvascular damage as a result of hyperglycaemia remains unclear [4, 5]. A number of interconnecting biochemical pathways have been proposed as potential links between hyperglycaemia and diabetic retinopathy. These include increased polyol pathway flux, activation of diacylglycerol- (DAG-)PKC pathway, increased expression of growth factors such as vascular endothelial growth factor (VEGF) and insulin-like growth factor-1 (IGF-1), haemodynamic changes, accelerated formation of advanced glycation endproducts (AGEs), oxidative stress, activation of the renin-angiotensin-aldosterone system (RAAS), and subclinical inflammation and leukostasis.

## 2. Polyol Pathway

**Figure 1:**
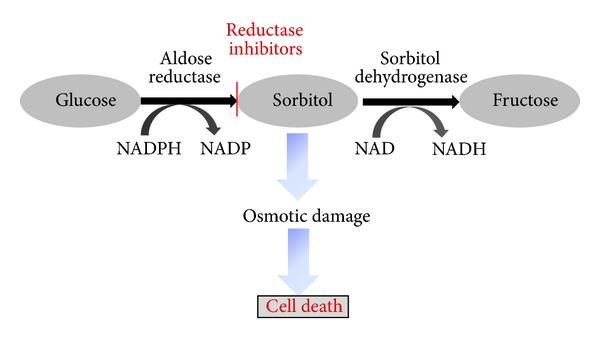
Polyol pathway.

In diabetes, the polyol pathway metabolises excess glucose (Figure 1). The enzyme aldose reductase (AR) present in the retina reduces glucose into sorbitol using nicotinamide adenine dinucleotide phosphate (NADPH) as a cofactor. Sorbitol is subsequently converted into fructose by sorbitol dehydrogenase (SDH). Since sorbitol is impermeable to cellular membranes, it accumulates within the cell, and this is followed by the slow metabolism of sorbitol to fructose [6, 7]. NADPH is also required for glutathione reductase as a cofactor for regenerating intracellular glutathione in cells, thus reducing the antioxidant capacity of the cells. 

The buildup of sorbitol is thought to have multiple damaging effects in retinal cells including osmotic damage [8]. In addition, the fructose produced by the polyol pathway can be phosphorylated to fructose-3-phosphate which in turn can be degraded to 3-deoxyglucosone, both of which are strong glycating agents and can result in the production of AGEs [9]. The use of NADPH as a cofactor in the polyol pathway results in less NADPH availability for use by glutathione reductase, which is crucial for the generation of reduced glutathione. This decrease in the reduced glutathione available results in a diminished protective response against oxidative stress [10]. The aberrant shift of the NADH/NAD+ ratio by SDH has been proposed to trigger NADH oxidase which can lead to the increased production of reactive oxygen species (ROS) within the cell [11].

Initial studies investigating the role of the polyol pathway in the pathogenesis of diabetic retinopathy were performed in diabetic animals fed with galactose [12–14]. These studies showed that aldose reductase inhibitors (ARIs) were able to reduce the incidence and severity of diabetic retinal lesions occurring in the galactose-fed animals. However, long-term studies (48 months) using galactose-fed dogs demonstrated that ARIs were not able to prevent vascular damage [15–17]. 

More recent studies have demonstrated that increased AR is localised in several retinal cells including pericytes [18, 19], retinal endothelial cells [20–22], ganglion cells [20, 23], Müller cells [20], retinal pigment epithelial cells [20], and neurons [23]. These studies also demonstrate that increased AR activity is involved in the destruction of retinal cells. Exposure of pericytes or endothelial cells to increased concentrations of glucose or galactose resulted in reduced viability of cells. However, this cell death was reversed upon the addition of ARIs [22–25].

The polyol pathway has also been implicated in several other pathophysiological changes which occur during diabetic retinopathy, one of these being the increased thickness of the retinal capillary basement membrane [26, 27]. Rat models of diabetes have shown that treatment with ARIs is able to prevent the thickening of the basement membrane [28, 29]. Another mechanism involved in the development of retinopathy is leukocyte adhesion to endothelial cells or leukostasis [30] as discussed later on in this paper. A study performed by Hattori et al. demonstrated that addition of an ARI to a diabetic rat model was able to attenuate the leukocyte adhesion to endothelial cells [31]. An increase in vascular permeability and the breakdown of the blood retinal barrier, hallmark processes which occur in diabetic retinopathy [32, 33] have been shown to be prevented by the application of ARIs [23, 34]. Genetic polymorphism studies also indicate that AR is involved in the development of diabetic retinopathy [35].

The administration of ARIs to animal model of diabetes at the onset of diabetes has demonstrated some benefit in preventing diabetic retinopathy [36–38]. However, ARI clinical trials, such as the sorbinil retinopathy trial, have shown little clinical benefit [38, 39]. It is thought that the poor performance of sorbinil was due to the insufficient inhibition of the polyol pathway in human tissue [40]. An ARI from a different structural class of drug, ARI-809, is highly selective and potent and seems to prevent retinopathy-like changes in an animal model of diabetes studies [40], but has not been tested in humans. Recent evidence suggests that the inhibition of both sorbitol and fructose is required to achieve beneficial effects in diabetic retinopathy [41]. More recently, the focus has also shifted to the potential role of SDH in diabetic retinopathy. Some workers have actually proposed that SDH is more important than AR in the development of diabetic retinopathy [42]. Targeted overexpression of SDH in retinal pericytes leads to toxicity via increased ROS production [43]. SDH also appears to be a genetic factor in diabetic retinopathy [44]. 

## 3. Nonenzymatic Protein Glycation

**Figure 2:**
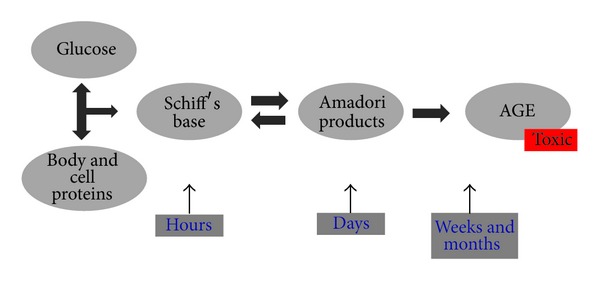
Formation of advanced glycation endproducts (AGEs).

Among the several pathogenic mechanisms that may contribute to diabetic retinopathy are the formation and accumulation of AGEs [45–47]. AGEs form at a constant but slow rate in the normal body starting at the embryonic development and accumulate with time. However, their formation is markedly accelerated in diabetes because of the increased availability of glucose [48]. AGEs are a heterogeneous group of molecules formed from the nonenzymatic reaction of reducing sugars with free amino groups of proteins, lipids, and nucleic acids. The initial product of this reaction is called a Schiff base, which spontaneously rearranges itself into an Amadori product [49] (Figure 2). A key characteristic of certain reactions or precursor AGEs is their ability for covalent crosslink formation between proteins, which alter their structure and function, as in cellular matrix, basement membranes, and vessel-wall components. Other major features of AGEs relate to their interaction with a variety of cell-surface AGE-binding receptors, including receptor for advanced glycation endproducts (RAGEs), leading to cellular activation and prooxidant, proinflammatory events [45]. AGEs affect cells by three main mechanisms: (1) as adducts occurring on modified serum proteins, (2) as endogenous adducts formed as a consequence of glucose metabolism, or (3) as extracellular matrix-immobilised modifications of long-lived structural proteins [50].

AGEs are important pathogenic mediators of almost all diabetic complications for instance, AGEs are found in retinal vessel of diabetic patients, and their levels correlate with those in the serum as well as with severity of retinopathy [50]. The interaction of AGEs with specific cell surface receptors has been implicated in the development of diabetic retinopathy [50]. These AGE receptors include the RAGE, galectin-3, CD36, and the macrophage scavenger receptor [50]. 

Intracellular production of AGE precursors involves the nonenzymatic reaction of reducing sugars with free amino groups of proteins, lipids, and nucleic acids. Early glycation and oxidation results in the formation of Schiff base which spontaneously rearranges itself into an Amadori product. Further glycation of proteins and lipids causes molecular rearrangements that lead to the generation of AGEs. 

There is evidence that there are three carbohydrate-derived oxidation products that are increased in diabetes: N^*ε*^-(carboxymethyl)lysine (CML), N^*ε*^-(carboxymethyl)hydroxylysine (CMhL), and pentosidine [51]. The increased concentrations of CML, CMhL and pentosidine in diabetes provides indirect evidence for a diabetes-related increase in oxidative damage to the protein [52]. CML, and CMhL are formed by oxidative cleavage of Amadori adducts, whereas pentosidine is a fluorescent cross-link formed between lysine and arginine residues in protein [53]. It has been demonstrated that the accumulation of CML in the neural retina and its associated vasculature shows a marked increase during diabetes [51]. During the development of diabetic retinopathy, the enzymatic and nonenzymatic pathogenic mechanisms proceed simultaneously and perhaps synergistically, suggesting that altered mitochondrial function and resultant oxidative stress may exacerbate both tissue damage and AGE formation [54]. The important role of oxidative stress in the pathogenesis of diabetic retinopathy is discussed in the latter part of this section. 

There is evidence from animal studies that exposure to high levels of AGEs contributes to renal and vascular complications [54, 55]. In a study by Hammes et al. [45], 26 weeks after the development of diabetes in rats, the retinal capillaries showed an increased accumulation of AGEs and loss of pericytes. Treatment with aminoguanidine (pimagedine) hydrochloride, AGE formation inhibitor significantly reduced AGE accumulation and prevented the formation of microaneurysms, acellular capillaries, and pericytes loss. Another study by Stitt et al. [54] showed that after 29 weeks of diabetes in rats, acellular capillaries were increased more than threefold together with a thickening of the basement membrane. Treatment with vitamin B6 derivative pyridoxamine, an inhibitor AGE formation, protected against capillary dropout and limited the production of basement membrane proteins. These early-stage experimental works suggest that AGE formation and activation of AGE receptors represent important, interconnected pathogenic mechanisms in diabetic retinopathy, and inhibition of these pathways presents a valid avenue for therapeutic exploitation [50].

Instead of reducing the accumulation of AGE products, a novel approach has been to design drugs with potential to break the AGE crosslinks. The recent stable and potent AGE-crosslink breaker is ALT-711 (now known as Alagebrium) that has been shown to reduce AGE-derived cross-links *in vivo* [56]. However, the effectiveness of ALT-711 to prevent the onset and progression of retinopathy remains to be investigated.

## 4. Protein Kinase C (PKC) Activation

**Figure 3:**
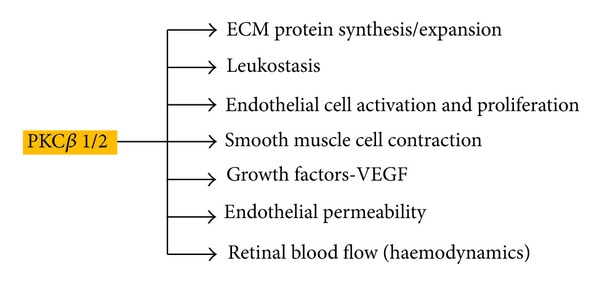
Regulation of pathophysiological processes in diabetic retinopathy by protein kinase C (PKC).

Protein kinase C (PKC) is a family of 10 enzymes, in which the *β*1/2 isoform appears to be closely associated with the development of diabetic retinopathy [57]. PKC is a serine/threonine kinase involved in signal transduction events responding to specific hormonal, neuronal, and growth factor stimuli. Hyperglycaemia leads to an increase in glucose flux through the glycolysis pathway, which in turn increases de novo synthesis of diacylglycerol (DAG), the key activator of PKC in physiology [58]. Clinical as well as experimental studies have highlighted an increase in expression of DAG and PKC activation in the diabetic state [59]. PKC is an important molecule in the regulation of numerous physiological processes. As a result, the activation of this enzyme has a cascade-like effect on several other pathways which in turn influence changes in endothelial permeability, retinal haemodynamics, and expression of vascular endothelial growth factor (VEGF) in the retinal tissue as well as increased activation and adhesion of leukocytes (leukostasis) [59–61] (Figure 3). 

Hyperglycemia increases de novo synthesis of diacylglycerol, which is an activating factor for the isoforms of protein kinase C. This activation in turn regulates various pathophysiological processes. 

The expression of the PKC *β*1/2 isoform is enhanced in patients with diabetes. Since PKC is involved in a number of physiological processes, its upregulation contributes to the pathogenesis of diabetic retinopathy in the form of differential synthesis of extracellular matrix (ECM) proteins and ECM remodelling, enhanced release of angiogenic factors, endothelial and leukocyte dysfunction leading to capillary occlusion and leukostasis, and changes in blood flow to the retina. As a result the PKC pathway directly influences other pathways such as inflammation, neovascularisation, and aberration of haemodynamics, which in turn further contribute to the pathogenesis and progression of diabetic retinopathy.

Investigations using LY333531 (ruboxistaurin mesylate, RBX), a specific inhibitor of PKC-*β*1/2, reported encouraging results in reducing leukostasis in diabetic patients [62]. RBX is a well-tolerated drug and has been tested extensively for the treatment of diabetic retinopathy in both experimental models as well as clinical trials. Experimental diabetes investigations suggest that RBX significantly reduced the progression of diabetic retinopathy [63]. This drug has undergone phase 3 clinical trials in the PKC diabetic retinopathy study (PKC-DRS, 2005) and the Diabetic Macular Edema Study (PKC-DMES, 2007). These studies reported that although PKC inhibition did not prevent diabetic retinopathy, it significantly reduced the loss of vision [64]. While the versatile action of PKC on the pathogenic pathways in diabetic retinopathy makes it an ideal therapeutic target, one must appreciate that the inhibition of this pathway would disrupt physiological pathways as well. Consequently, despite success at some clinical trials and few significant adverse effects, RBX has not translated into therapy for diabetic retinopathy. However, clinical trials have suggested that the drug may reduce visual loss, and the need for focal laser.

## 5. Haemodynamic Changes

The Wisconsin Epidemiological Study of Diabetic Retinopathy (WESDR) as well as the UKPDS have reported the significant role of blood pressure in the progression of PDR [65–67]. It is also well documented that patients with diabetes have a high incidence of hypertension [68, 69]. Moreover, a study by Patel et al. [70] demonstrated that, following successful photocoagulation, blood flow to the retina was found to be lowered; this was interpreted as a correction in haemodynamic autoregulation.

Hypertension is thought to contribute to the progression of diabetic retinopathy through two mechanisms. Firstly, the mechanical stretch and sheer stress imparted on endothelial cells by high blood pressure and increased perfusion of the retina, as well as higher viscosity of the blood, lead to endothelial dysfunction [71]. Secondly, the endocrine system involved in blood pressure regulation is also independently implicated in the pathogenesis of diabetic retinopathy [72].

## 6. Renin-Angiotensin-Aldosterone System

The renin-angiotensin-aldosterone system (RAAS) is the endocrine system that plays an important role in the regulation of blood pressure and fluid balance and shows an aberration in patients with diabetes [72]. The expression of the receptors and signalling molecules of RAAS, namely, renin, angiotensin converting enzymes I and II (ACEI and ACE II), as well as angiotensin receptors, has been reported to increase in the retina, during PDR [72, 73]. This change in expression has been found independent of systemic blood pressure. Studies on experimental models provide evidence that ACE inhibition prevents neovascularisation, a hallmark feature of early diabetic retinopathy [74]. Large clinical studies have also compared angiotensin receptor blockers (ARBs), candesartan, and ACE inhibitors as an alternative therapeutic target in the RAAS pathway. The Diabetic Retinopathy Candesartan Trials (DIRECT) and Renin Angiotensin System Study (RASS) both reported a reduction in the incidence of retinopathy in Type 1 diabetes at baseline as well as a reduction in the progression of retinopathy [75–77]. 

Although the precise mechanism by which RAAS contributes to diabetic retinopathy is not well elucidated, *in vitro* studies have suggested that angiotensin II is involved in the PKC activation as well as VEGF signaling [78]. These studies provide new insight into the significance of RAAS blockade as a novel therapy for diabetic retinopathy. 

## 7. Subclinical Inflammation and Leukostasis

Studies such as the Hoorn Study have reported and highlighted the important role of subclinical inflammation in the development of diabetic retinopathy [79–81]. It is now established that the role of inflammation in diabetic retinopathy is both prominent as well as complex. While hyperglycaemia, oxidative stress, advanced glycation endproduct formation, and hypertension all contribute to inflammation, the inflammatory response itself propagates these pathways further, through cytokines, adhesion molecules, VEGF signalling, enhanced RAGE expression, changes in nitric oxide regulation and NF-*κ*B signalling. Therefore, subclinical inflammation in the retina leads to increased intraocular blood pressure via endothelial nitric oxide synthase (eNOS), the formation of new, weak vessels and their increased permeability due to VEGF which in turn leads in haemorrhages in the retina, and leukostasis due to the cross talk between several proinflammatory factors. Leukostasis is an important event in diabetic retinopathy pathogenesis, leading to capillary occlusion and ROS-mediated cell death, as well as amplifying the inflammatory response locally in the retinal tissue [82–85].

It is well reported that there is a significant increase in the systemic expression of proinflammatory cytokines, activation of soluble and cell surface adhesion molecules, and the expression of chemokines in the retina of patients with diabetic retinopathy [86]. Several clinical studies provide evidence that this increase in serum proinflammatory cytokines, adhesion molecules, and the activation of immune cells in the diabetic state correlates with the progression of diabetic retinopathy [87, 88]. It is well accepted that endothelial dysfunction and increased levels of proinflammatory cytokines and adhesion molecules contribute to leukostasis by enhancing leukocyte and endothelial cell interaction [89, 90]. This was further confirmed by studies wherein knocking out adhesion molecules greatly reduced levels of leukocyte entrapment in retinal capillaries in experimental models [91]. 

Recent studies have shown that changes in the expression of carbohydrate chains on the surface of leukocytes lead to leukocyte activation and greatly enhance leukocyte rolling and adhesion to endothelial cells. The inflammatory enzyme UDP-GlcNAc:Gal*β*1-3GalNAc*β*-R-*β*-1-6-N-acetylglucosaminyltransferase (C2GNT) shows higher activity in diabetic patients. This hyperglycaemia and tumour-necrosis-factor-alpha-(TNF-*α*-) induced enzyme bring about increased O-glycosylation type modifications on the surface carbohydrate chains of leukocytes, leading to leukocyte dysfunction and increased leukostasis [84, 92]. The activity of the enzyme has been correlated with the progression and severity of diabetic retinopathy as well as neuropathy [30, 86].

Local inflammation involving the activation of microglia, resident macrophages, and immune cells is also thought to play a role in the pathogenesis of diabetic retinopathy [93]. The activation of microglia in the diabetic retina can lead to the increased production of proinflammatory cytokines, ROS, growth factors, matrix metalloproteinases (MMPs), and nitric oxide all of which have been implicated in the pathogenesis of diabetic retinopathy [94]. The role of microglia activation has been supported by the use of minocycline, an antibiotic and anti-inflammatory agent, to block the activation of microglia and prevent diabetic retinopathy [93]. 

Several other therapeutic approaches targeting inflammation have been studied in both animal models and clinical trials in recent years. The use of anti-inflammatory drugs such as the intravitreal triamcinolone acetonide (IVTA) and nonsteroidal anti-inflammatory drugs such as nepafenac has been reported to reduce VEGF expression, normalise vascular permeability, reduce levels of cell death and leukostasis, and improve visual acuity [95–97]. It must be noted, however, that these drugs appeared to have a significant impact during later stages and failed to prevent early development or progression of diabetic retinopathy. Side effects associated with mode of delivery of these drugs, namely, intravitreal injections, which include glaucoma, pseudoendophthalmitis, and endophthalmitis are also highly undesirable [96]. Consequently there is a great deal of interest in the development of topically administered and intraocular implants for the delivery of such anti-inflammatory steroids [98]. Anti-TNF-*α* antibody ESBA105 has also been studied with topical administration on animal models and has been reported to have a positive impact on reducing neovascularisation [99]. Infliximab, a TNF-*α* antagonist, has undergone phase III clinical trials with success in reducing macular thickness and generally improving diabetic vision [93]. 

On the evidence from clinical studies, aspirin has shown only little or no benefit in preventing diabetic retinopathy [99]. However, further work is still needed to test if high dose aspirin is useful as a preventive treatment in diabetic retinopathy.

## 8. Oxidative Stress

**Figure 4:**
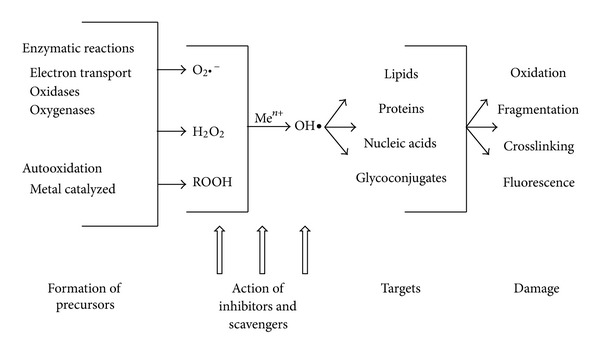
Pathway showing the effect of oxidative stress on the development of diabetic complications adapted from [100].

Oxidative stress may be defined as an imbalance between the level of ROS or oxygen radicals and the antioxidant defences in a biological system [100]. Oxidative stress and resultant tissue damage are hallmarks of chronic disease and cell death. A hypothetical sequence of events by which oxidative stress may be linked to tissue damage and the development of pathophysiology is outlined in Figure 4. ROS and reactive nitrogen species (RNS) are the two major types of oxidants.

Under normal physiological conditions, ROS are either detoxified by interaction with various reducing and sequestering agents including thioredoxin, glutathione (GSH), and tocopherol (vitamin E) or by enzymes such as superoxide dismutases (SODs), catalase, glutathione peroxidase, and thioredoxin reductase [101, 102]. Oxidative stress induced by hyperglycaemia is an important pathway of diabetic microvascular complications [103], and increasing evidence suggests that the correlation between hyperglycaemia, changes in the redox homeostasis, and oxidative stress is the key event in the pathogenesis of diabetic retinopathy [104–106]. It has been hypothesised that both the development and the progression of retinopathy result from increased oxidative species [107, 108].

The scheme in Figure 4 highlights the various enzymatic reactions that lead to the formation of sources of ROS. These species then target macromolecules causing chemical modification of these biological molecules thus causing damage to the cell and tissue functions, leading to pathology. Inhibitors and scavengers of ROS can limit the increased accumulation of these reactive species.

Increased oxidative stress may result from over production of precursors to reactive oxygen radicals and/or decreased efficiency of inhibitory and scavenger systems [100]. Animal studies have demonstrated that oxidative stress contributes not only to the development of retinopathy but also to the resistance of retinopathy to reverse after good glycaemic control is reinstituted [109]. Superoxide levels are elevated in the retina of diabetic rats and in retinal cells incubated in high glucose media and hydrogen peroxide content which is increased in the retina of diabetic rats, and membrane lipid peroxidation and oxidative damage to DNA (as a consequence to ROS-induced injury) are elevated in the retina in diabetes [110, 111]. In diabetes the activities of antioxidant defence enzymes responsible for scavenging free radicals and maintaining redox homeostasis such as SOD, glutathione reductase, glutathione peroxidase, and catalase are diminished in the retina [112, 113]. Animal studies have provided evidence for and against the use of antioxidants to prevent experimental diabetic retinopathy [112, 113]. Although the use of antioxidants has shown some benefit, this has not been supported by clinical trials.

Brownlee and colleagues have suggested that oxidative stress may represent a “unifying mechanism” which links all of the damaging biochemical pathways induced by hyperglycaemia in diabetic retinopathy [114]. They propose that mitochondrial-derived ROS causes strand breaks in DNA which in turn activates poly-(ADP-ribose)-polymerase (PARP). The activation of PARP inhibits glyceraldehyde phosphate dehydrogenase (GAPDH) activity which causes the accumulation glycolytic of metabolites. The metabolites then activate the AGE, PKC*β*2, polyol, and hexosamine pathways [114]. Other studies have suggested the important role of NADPH oxidase-derived ROS in the pathogenesis of diabetic retinopathy [114].

Recent evidence has implicated ROS-mediated activation of metalloproteinase-2 (MMP-2) in the development of diabetic retinopathy [115]. The activation of MMP-2 is thought to cause cell death of retinal endothelial cells by causing mitochondrial dysfunction which activates the apoptosis cascade. 

## 9. Growth Factors

The involvement of growth factors in diabetic retinopathy is supported by the clinical evidence of the development of retinopathy during puberty and the observation that serious retinopathy is rarely observed in growth hormone deficient diabetic dwarfs [116]. In addition studies performed in the 1970s showed that pituitary ablation slowed the progression of diabetic retinopathy [117, 118]. 

There are a number of growth factors which have been associated with the development of diabetic retinopathy and these include basic fibroblast growth factor (bFGF) [119], insulin-like growth factor-1 (IGF-1) [120, 121], angiopoietin-1 and -2 [122–124], stromal-derived factor-1 [125], epidermal growth factor (EGF) [126], transforming growth factor-beta 2 (TGF-*β*2) [127], platelet-derived growth factors (PDGFs) [128], and erythropoietin [129]. 

The insulin-like growth factors (IGFs) are produced by the majority of body tissues and are mediators of cell growth, differentiation, and transformation. Increased levels of IGF-1 have been found in the vitreous fluid and serum of diabetic patients [130, 131]. The precise role of IGF in diabetic retinopathy pathogenesis remains unknown. However, increasing evidence suggests that IGFs work directly within target tissues as well as systemically [131, 132]. A number of studies also suggest that the action of IGF in neovascularisation is controlled by vascular endothelial growth factor (VEGF) [133].

**Figure 5:**
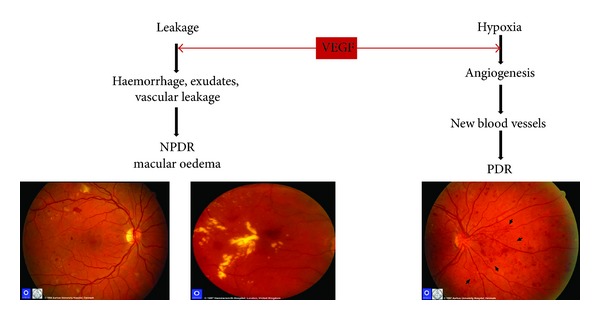
Vascular endothelial growth factor (VEGF) pathways in nonproliferative diabetic retinopathy (NPDR) and proliferative diabetic retinopathy (PDR) and carbonic anhydrase (CA).

The growth factor which is the most widely studied in relation to diabetic retinopathy is VEGF which exists in four homodimeric molecular species, each monomer having, respectively, 121, 165, 189, or 206 amino acids [134]. VEGF promotes angiogenesis; causes breakdown of the blood-retinal barrier, stimulation of endothelial cell growth, and neovascularisation; and increases vascular permeability in the ischemic retina [135–137] (Figure 5). Many animal and clinical studies performed have established a role for VEGF, particularly the 165 isoform, in the development and progression of diabetic retinopathy [138, 139]. The cellular functions of VEGF are mediated by the activation of two membrane bound tyrosine kinase receptors [140]. The binding of VEGF to the membrane bound receptors activates two possible pathways, a calcium influx channel or a mitogen activating protein kinase signalling pathway. Both pathways lead to the vascular leakage and blood retinal barrier breakdown with which VEGF has been associated [141]. The angiogenic role in the retina to which VEGF has been linked is thought to be due to an interaction with angiotensin II [142]. VEGF has been linked with leukocyte adhesion to retinal endothelial cells this is thought to occur via induction of nitric oxide synthase and intracellular adhesion molecule-1 expression [143]. 

The discovery of the role of VEGF in diabetic retinopathy has led to the development of anti-VEGF agents as therapy for the treatment of diabetic complications. The current anti-VEGF agents include pegaptanib, ranibizumab, and bevacizumab [144–149]. Recent development in the use of anti-VEGF drugs includes the VEGF-trap (Regeneron, Tarrytown, NY, US), a recombinant fusion protein against VEGF-A, and placental growth factor, for diabetic retinopathy. Early clinical results suggest efficacy for the treatment of DMO [143].

While anti-VEGF drugs are often effective, they do not work in all patients. In addition, recent evidence has suggested caution in the long-term use of anti-VEGF drugs for diabetic retinopathy. Since VEGF has a role as a retinal neuron survival factor, there appears to be a link between the use of anti-VEGF drugs and the death of cells (photoreceptors and Muller glia) involved in visual function [143].

## 10. Carbonic Anhydrase

The increased intraocular level of VEGF correlates to the increase in vascular permeability which contributes to haemorrhage, exudates, and vascular leakage leading to NPDR and angiogenesis and vasculogenesis leading to PDR. 

Carbonic anhydrases (CAs) are a group of ubiquitous metalloenzymes, which function by causing rapid conversion of carbon dioxide to bicarbonate and protons. There have been 4 isoforms of carbonic anhydrase identified to be present in the eye in various cell types [150]. In a recent study by Gao et al. it was shown that diabetic patients had significantly higher concentrations of CA than healthy controls [151]. It has been demonstrated that CA inhibitors including acetazolamide and benzolamide can reduce the progression of diabetic retinopathy and prevent visual loss in both animal and clinical studies [150]. The mechanisms which have been suggested by which CA inhibitors can have beneficial effect in diabetic retinopathy are reducing humour secretion, inducing vasodilatation and improving blood flow to the ocular region, inhibiting platelet aggregation and reducing vascular permeability [151].

## 11. Retinal Neurodegeneration

It is now widely accepted that, in addition to the vascular changes, structural and functional damage to nonvascular cells (ganglion cells, glial cells, microglial) contributes to the pathogenesis of diabetic retinopathy [152–155]. There is evidence suggesting that neurodegeneration of retinal neurons and glial cells occurs even before the development of microaneurysms [156].

## 12. Conclusion

Diabetic retinopathy remains the leading cause of blindness and visual impairment in the working age population. The pathophysiology of diabetic retinopathy has been extensively studied and many contributing biochemical pathways have been identified. Although it is unlikely that a cure for diabetic retinopathy would be identified, continual research aimed at providing a better understanding of these mechanisms is helping in the development of novel therapeutic agents for the effective treatment of diabetic retinopathy. 

## Non-Standard Abbreviations

ROS: Reactive oxygen species
NPDR: Non proliferative diabetic retinopathy
PDR: Proliferative diabetic retinopathy
ECM: Extracellular matrix
O_2_^−^: Superoxide radical
MMPs: Matrix metalloproteinases
SOD: Superoxide dismutase
AGE: Advanced glycation endproduct
RAAS: Renin-angiotensin-aldosterone system
PKC: Protein kinase C
DCCT: Diabetes Control and Complications Trial
UKPDS: United Kingdom Prospective Diabetes Study
VEGF: Vascular endothelial growth factor
IGF-1: Insulin-like growth factor-1
DMO: Diabetic macular oedema
NADPH: Nicotinamide adenine dinucleotide phosphate
SDH: Sorbitol dehydrogenase
AR: Aldose reductase
ARIs: Aldose reductase inhibitors
RAGE: Receptor for advanced glycation endproducts
CMhL: N^*ε*^-(carboxymethyl)hydroxylysine
CML: N^*ε*^-(carboxymethyl)lysine
ECM: Extracellular matrix
WESDR: Wisconsin Epidemiological Study of Diabetic Retinopathy
DIRECT: Diabetic Retinopathy Candesartan Trials
RASS: Renin Angiotensin System Study
eNOS: Endothelial nitric oxide synthase
PARP: Poly-(ADP-ribose)-polymerase
GAPDH: Glyceraldehyde phosphate dehydrogenase
MMPs: Metalloproteinases
IVTA: Intravitreal triamcinolone acetonide
EGF: Epidermal growth factor
TGF-*β*2: Transforming growth factor-beta 2
PDGFs: Platelet-derived growth factors
PKC-DRS: PKC diabetic retinopathy study
PKC-DMES: Diabetic Macular Edema Study
CAs: Carbonic anhydrases
GSH: Glutathione
TNF-*α*: Tumour necrosis factor-alpha
DAG: Diacylglycerol.
